# Pathogen dynamics under both bottom‐up host resistance and top‐down hyperparasite attack

**DOI:** 10.1111/1365-2664.13185

**Published:** 2018-06-19

**Authors:** Steven R. Parratt, Anna‐Liisa Laine

**Affiliations:** ^1^ Research Centre for Ecological Change University of Helsinki Helsinki Finland

**Keywords:** *Ampelomyces* spp., bottom‐up, disease biology, hyperparasite, plant pathogen, *Plantago lanceolata*, *Podosphaera plantaginis*, top‐down

## Abstract

1. The relative importance of bottom‐up versus top‐down control of population dynamics has been the focus of much debate. In infectious disease biology, research is typically focused on the bottom‐up process of host resistance, wherein the direction of control flows from the lower to the higher trophic level to impact on pathogen population size and epidemiology. However, the importance of top‐down control by a pathogen's natural enemies has been mostly overlooked.
2. Here, we explore the effects of, and interaction between, host genotype (i.e., genetic susceptibility to pathogen infection) and infection by a hyperparasitic fungus, *Ampelomyces* spp., on the establishment and early epidemic growth and transmission of a powdery mildew plant pathogen (*Podosphaera plantaginis*). We used a semi‐natural field experiment to contrast the impacts of hyperparasite infection, host‐plant resistance and spatial structure to reveal the key factors that determine pathogen spread. We then used a laboratory‐based inoculation approach to test whether the field experiment results hold across multiple pathogen–host genetic combinations and to explore hyperparasite effects on the pathogen's later life‐history stages.
3. We found that hyperparasite infection had a negligible effect on within‐host infection development and between‐host spread of the pathogen during the onset of epidemics. In contrast, host‐plant resistance was the major determinant of whether plants became infected, and host genotype and proximity to an infection source determined infection severity.
4. Our laboratory study showed that, while the interaction between host and pathogen genotypes was the key determinant of infection outcome, hyperparasitism did, on average, reduce the severity of infection. Moreover, hyperparasite infection negatively influenced the production of the pathogen's overwintering structures.
5. *Synthesis and applications*. Our results suggest that bottom‐up host resistance affects pathogen spread, but top‐down control of powdery mildew pathogens is likely more effective against later life‐history stages. Further, while hyperparasitism in this system can reduce early pathogen growth under stable laboratory conditions, this effect is not detectable in a semi‐natural environment. Considering the effects of hyperparasites at multiple points in pathogen's life history will be important when considering hyperparasite‐derived biocontrol measures in other natural and agricultural systems.

## Abstract 2

Our results suggest that bottom‐up host resistance affects pathogen spread, but top‐down control of powdery mildew pathogens is likely more effective against later life‐history stages. Further, while hyperparasitism in this system can reduce early pathogen growth under stable laboratory conditions, this effect is not detectable in a semi‐natural environment. Considering the effects of hyperparasites at multiple points in pathogen's life history will be important when considering hyperparasite‐derived biocontrol measures in other natural and agricultural systems.

## 1 INTRODUCTION

The potential for unchecked infectious disease outbreaks is one of the greatest threats to the stability and persistence of natural, agricultural and human populations. We, therefore, need robust conceptual models that incorporate all the factors that govern the establishment, growth and transmission of pathogens to effectively predict and counter disease spread. However, despite the ubiquity of pathogens in nature, we lack a thorough understanding of the ecological factors that limit their growth and transmission, and thus moderate their impacts upon host populations. Recently, there has been a push to study infectious diseases within an ecological framework that accounts for all of the interactions pathogens are engaged in (Johnson, de Roode & Fenton, 2015; Pedersen & Fenton, 2007). Indeed, it has become increasingly apparent that in addition to the obligate interaction with their host, pathogens often interact with a suite of coinfecting symbionts, with pronounced consequences for infection development, spread, virulence and evolution (Alizon & van Baalen, 2008; May & Nowak, 1995). However, considering only bottom‐up control through host resistance, and intraguild competition among coinfecting pathogens, potentially overlooks a third crucial limiting factor to disease progression; top‐down regulation by natural enemies. Given that most pathogens are likely to be under attack by predators and are open to infection by hyperparasites (Parratt & Laine, 2016), top‐down control in natural systems may be an important determinant of disease success.

In the broadest sense, host resistance to infection operates as bottom‐up control because the direction of influence flows from the lower to the higher trophic level by either actively or passively limiting a pathogen's access to resources. Indeed, the efficacy of host resistance in controlling disease underpins current theories of host–pathogen coevolution (Frank, 1991; Grenfell & Dobson, 1995; Lively, 2010). Empirical studies show that variation in host resistance can strongly influence pathogen establishment and transmission in natural populations (Susi & Laine, 2015), and this effect is reflected in patchwork patterns of pathogen incidence (Jousimo et al., 2014; Laine, Burdon, Dodds & Thrall, 2011; Springer, 2007). Moreover, breeding of resistant host genotypes and immune mobilisation through vaccination are components of our current disease control efforts, both of which conceptually rely on host‐derived, bottom‐up suppression of pathogens (McManus, Paim, Melo, Brasil & Paiva, 2014; Zhan, Thrall & Burdon, 2014). However, resistance alone is rarely the sole determinant of infection outcome (Price, Bever & Clay, 2004) and is often sensitive to external factors. For instance, the abiotic environment can play a key role in determining infection outcome in both plant (Barrera, Hoy & Li, 2012; Laine, 2007; Tack, Laine, Burdon, Bissett & Thrall, 2015) and animal (Blanford, Thomas, Pugh & Pell, 2003; Mitchell, Rogers, Little & Read, 2005) systems. Furthermore, biotic processes such as protective symbionts (Ford, Kao, Williams & King, 2016) and behavioural self‐medication (Milan, Kacsoh & Schlenke, 2012) have been shown to limit the negative effects of parasite infection. Thus, considering the effect of host resistance in isolation will almost certainly give an incomplete picture of factors determining disease dynamics.

In contrast to bottom‐up host resistance, top‐down control of disease by natural enemies such as hyperparasites or predators is poorly understood. Generally, considering any infectious agent (parasite or hyperparasite) as a source of top‐down, regulatory control has received only limited empirical attention (Albon et al., 2002; Redpath, Mougeot, Leckie, Elston & Hudson, 2006). Indeed, the most robust evidence for such a phenomenon comes from studies of parasitoids and hyperparasitoids (e.g., Schooler, de Barro & Ives, 2011), organisms that occupy an ecological niche somewhere between predators and true pathogens (Godfray, 1994), and the effects of predators on invertebrate plant pests (Schellhorn, Bianchi & Hsu, 2014). Some theoretical and mostly observational research supports the hypothesis that hyperparasite infection may influence pathogen population structure and dynamics (Andersen et al., 2012; Holt & Hochberg, 1998; Springer, Baines, Fulbright, Chansler & Jarosz, 2013; Tollenaere et al., 2014). Probably the best studied case is the hypovirulence‐inducing mycovirus CHV‐1, which infects the chestnut blight pathogen *Cryphonectria parasitica*. CHV‐1 reduces the pathogen's virulence (Nuss, 2005) and consequently has cascading effects on populations of the chestnut tree base‐host (Davelos & Jarosz, 2004; Morozov, Robin & Franc, 2007). When introduced, this hyperparasite is able to spread throughout pathogen populations (Prospero & Rigling, 2016), but its broad use as a biocontrol agent is hampered because, among other reasons, it is easily outcompeted by other viral isolates (Robin, Lanz, Soutrenon & Rigling, 2010). However, in most systems, we lack evidence for hyperparasite effects on within‐host–pathogen growth and between‐host transmission, particularly in a natural context. Understanding this is nontrivial because this assumption lies at the heart of using hyperparasites as biocontrol agents in agriculture and medicine (Kiss, Russell, Szentiványi, Xu & Jeffries, 2004; Nobrega, Costa, Kluskens & Azeredo, 2015). Given that hyperparasitism is likely common in nature (Parratt & Laine, 2016), it is also likely to exert a major influence on natural pathogen populations.

The relative importance of bottom‐up and top‐down regulation in natural communities (Slobodkin, 1961) has received considerable attention in the literature. Evidence for the importance of either process is somewhat divided, depending on whether one examines terrestrial or aquatic habitats (Heath, Speirs & Steele, 2014), the productivity of the biological system (Borer, Halpern & Seabloom, 2006), or the complexity of the food chains involved (Mooney et al., 2010). Pedersen and Fenton (2007) have extended this framework to consider host immunity as both top‐down and bottom‐up control, to distinguish between host resource limitation and active suppression of infection. However, in the strictest trophic‐level view, the notion that pathogens themselves are under simultaneous top‐down and bottom‐up control has mostly been explored in the context of virulence evolution in the face of predation and host resistance (Friman, Ghoul, Molin, Johansen & Buckling, 2013). To date, the potential for interactions between top‐down control by hyperparasite and bottom‐up control by host resistance has not been studied. We, thus, do not know how these factors combine to affect key pathogen life‐history stages in ecological time and how this might influence disease dynamics.

Here, we aim to determine how bottom‐up host resistance and top‐down hyperparasite attack interact to alter the infection dynamics of the powdery mildew pathogen *Podosphaera plantaginis*. We focus our study on the establishment and onset of epidemics because biocontrol strategies are likely to be most valuable when they curtail or halt the incipient spread of disease. Natural populations of the pathogen's obligate host, *Plantago lanceolata*, vary in both their quantitative and qualitative resistance to local pathogen strains. This variation in resistance shapes the strength of epidemics at the local scale in nature and, in turn, affects the metapopulation dynamics of the pathogen (Jousimo et al., 2014; Susi & Laine, 2015). *Podosphaera plantaginis* is also subject to frequent attack by the fungal hyperparasite *Ampelomyces* (Parratt, Barres, Penczykowski & Laine, 2016), and previous work has found a negative impact of hyperparasitism on pathogen overwinter survival (Tollenaere et al., 2014). To date, there is some evidence that *Ampelomyces* mycoparasites inhibit growth and transmission of other powdery mildew species (Kiss et al., 2004), and thus represent a putative source of top‐down control on *Po. plantaginis* population dynamics.

We first use a semi‐natural experiment to simulate the impact of the hyperparasite and host resistance on pathogen spread early in the epidemic season. We test (a) the effect of hyperparasite infection on within‐host growth of the pathogen at initial infection foci and (b) how this interacts with host genotype and spatial structure to influence transmission of the pathogen between host individuals during the onset of epidemics. We then use laboratory experiments to determine (c) how host‐plant genotype and *Ampelomyces* infection impact key life‐history stages of several distinct pathogen strains.

## 2 MATERIALS AND METHODS

### 2.1 Study system

*Podosphaera plantaginis* is a specialist obligate fungal pathogen which causes powdery mildew symptoms on *Pl. lanceolata. Podosphaera plantaginis* grows on the above‐ground tissues of its host as radial lesions of hyphae that support chains of asexual spores (conidia). Mature conidia are wind‐dispersed to neighbouring plants where they instigate fresh pathogen growth. The rate of growth and spore production is sensitive to environmental conditions; infections appear slowly at focal overwintering sites in late spring before epidemic spread accelerates later in the summer (Laine, 2004). Common‐garden experiments have found that infected plants begin transmitting spores at *c*. 30 days post infection (dpi; Susi, Barres, Vale & Laine, 2015), reaching peak transmission at 40–50 dpi, although this is likely to vary with environmental conditions. *Podosphaera plantaginis* survives overwinter in structures called chasmothecia, which persist in the leaf litter and initiate new infections in the following spring/summer as the host regrows (Tollenaere & Laine, 2013). *Podosphaera plantaginis* and its host have a coevolutionary relationship, wherein the host has evolved quantitative and qualitative resistance to the pathogen, which prevent pathogen establishment and limit spore transmission, respectively (Susi et al., 2015; Tack, Thrall, Barrett, Burdon & Laine, 2012).

*Podosphaera plantaginis* is host to the hyperparasite *Ampelomyces* spp., a species complex of fungi found infecting powdery mildew species worldwide (Kiss et al., 2004). Field surveys have shown that *Ampelomyces* infections are detectable early during mildew epidemics (Parratt et al., 2017 and Supporting Information Figure S1), and severely reduce overwinter survival of *Po. plantaginis* in nature (Tollenaere et al., 2014). In other mildew species, *Ampelomyces* reduces conidial production and reverses mildew‐derived tissue damage in hosts under controlled conditions (Abo‐Foul, Raskin, Sztejnberg & Marder, 1996; Falk, Gadoury, Pearson & Seem, 1995). However, the effects of *Ampelomyces* infection on *Po. plantaginis* in the field, and its influence on the critical onset phase of epidemics, when host resistance is an important factor, are unknown.

Isolation, culture conditions and selection criteria for the lines of plant, pathogen and hyperparasite used here are given in Supporting Information Table S1. The hyperparasite strain used in all experiments was previously identified as a broadly infective, rapidly growing strain: “294_11” (Parratt et al., 2017).

### 2.2 Host resistance and hyperparasite infection under semi‐natural conditions

**Figure 1:**
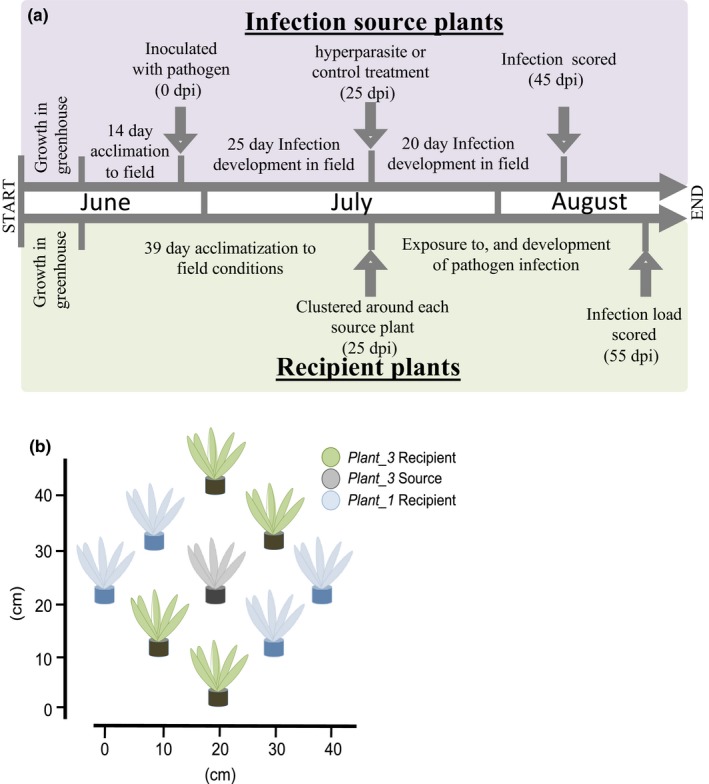
Schematic of the semi‐natural experiment workflow. (a) Timeline of experiment. Pathogen‐inoculated “infection source plants” (purple box) were used to test within‐host transmission of mildew under hyperparasite attack. “Recipient plants” (green box) were used to test among host transmission under hyperparasite attack, host resistance and host spatial structure. (b) Clusters consisted of either all *Plant_3* genotypes or a 1:1 mix of *Plant_3* and *Plant_1* genotypes

We conducted a semi‐natural experiment at Kumpula botanical gardens, University of Helsinki, Helsinki (60.203194, 24.956843) in the summer of 2015 to test: (1) if hyperparasite attack at incipient pathogen development can affect within‐host disease spread, and (2) if the spread of the pathogen among hosts during the onset of epidemics is most influenced by hyperparasite attack at the infection source or quantitative resistance in recipient plants (Figure 1a).

To test (1), we simulated the biology of the hyperparasite infecting newly emerging pathogen foci and scored pathogen development within these host plants. We inoculated two leaves on each of 22 six‐week‐old *Pl. lanceolata* clones of genotype *Plant_3* (called “source plants” hereafter) with a single powdery mildew isolate (*Pathogen_3*). Source plants were grown from clonal rootstock in greenhouse conditions for 4 weeks, then placed outside to acclimatise for 2 weeks prior to inoculation. *Podosphaera plantaginis* conidia of clonal strain *Pathogen_3* were inoculated onto two leaves per plant by evenly spreading spores across the leaf surface with a sterile paintbrush. Leaves were only inoculated if they were >10 cm long (Supporting Information Table S2). Inocula were from 14‐day‐old 1‐cm^2^ lesions, grown on susceptible host leaves. This method has been shown to produce repeatable infection outcomes (Laine, 2004). Inoculated leaves were marked with coloured string, so they could be identified later. Source plants were then kept in growth chambers (16 h:8 h light:dark, 20°C) for 48 hr to allow infection to establish. Plants were then moved to the field in their pots and kept at least 2 m apart to prevent cross‐contamination. Once mycelial pathogen growth was visible (25 dpi), both leaves that had received the pathogen were inoculated with *Ampelomyces* as a 70 ± 2 μl spray of spores in filter‐sterilised H_2_O at 1 × 10^6^ spore per ml on half of the source plants, while the other half received the same volume of filter‐sterilised H_2_O as a control. Cardboard funnels were used to ensure that the treatment was delivered only to the intended leaves. This time point was chosen as (a) *Ampelomyces* requires established mildew mycelia to infect, so this represents the earliest time point that a biocontrol application would be effective, and (b) previous studies have shown that transmission of mildew spores from infected plants is negligible before 30 dpi (Susi et al., 2015). Fungal infections were allowed to develop on source plants until 45 dpi, at which point the number of mildew‐infected leaves was scored. Up to 10 mildew‐infected leaves were removed and dried in paper envelopes for DNA extraction and qPCR screening for the hyperparasite (see Tollenaere et al., 2014 and Supplementary methods in Supporting Information Appendix S1). Under laboratory conditions, *Ampelomyces* infects mildew mycelia within a few hours of inoculation, rapidly invades the pathogen's mycelia, and produces spore structures within 3–6 days of infection (Kiss et al., 2004). Thus, this 20‐day period was assumed to allow time for the *Ampelomyces* to establish and exert any effect on the pathogen during the early epidemic stages that we are testing here.

To test (2): we wanted to directly compare the influence of hyperparasite attack at the source of infection and variation in host resistance among recipient plants during the onset of epidemics. We surrounded the source plants (22) with eight “recipient” plants on the same day as the *Ampelomyces* treatment (25 dpi). This timing was chosen to capture a biologically realistic effect of the hyperparasite infecting a newly emerging disease site in a population of potential host plants—a scenario that occurs in nature (Parratt et al., 2017 and Supporting Information Figure S1). Recipient plants were clones of either *Plant_3* or a second genotype, *Plant_1*, which preliminary experiments had shown to have similar qualitative, but differing quantitative resistance against *Pathogen_3*. Recipients were grouped around source plants at either 10 and 20 cm distances shown to represent significant spatial structure for this pathogen (Tack, Hakala, Petaejae, Kulmala & Laine, 2014), measured between the central rosettes. Clusters with both “recipient” genotypes had two clones of each line at each distance (Figure 1b). Previous work with *Po. plantaginis* under similar semi‐natural field conditions identified 40–50 dpi as the peak transmission window for mildew‐infected plants, and our recipients were exposed to infection sources during this window (Susi et al., 2015). Recipient plants remained in the field for 10 days after focal plants were removed, as this allowed powdery mildew symptoms to visibly develop but minimised autoinfection. We scored the establishment of powdery mildew and the number of infected leaves on recipient plants at 55 dpi. Up to 10 mildew‐infected leaves were removed and dried for DNA extraction and qPCR screening for the hyperparasite (see Supplementary methods in Supporting Information Appendix S1).

### 2.3 Combining host genotype and hyperparasite under laboratory conditions

We conducted a laboratory experiment to (a) confirm previous findings that mildew growth differs across host‐plant genotypes (Laine, 2004, 2007) and (b) to determine if any differences between host resistance and hyperparasitism in our semi‐natural experiment hold true for multiple pathogen genotypes. We challenged five strains of *Po. plantaginis* (*Pathogen_1*,* Pathogen_2*,* Pathogen_3*,* Pathogen_4* and *Pathogen_5*) growing on three clonal host‐plant genotypes (*Plant_1*,* Plant_2* and *Plant_3*) with either a single isolate of *Ampelomyces* (294_11) or a H_2_O control. Inoculations were carried out on detached *Pl. lanceolata* leaves placed onto moist filter paper in Ø 9 cm Petri dishes in a growth chamber (16 h:8 h light:dark, 20°C). *Podosphaera plantaginis* inoculation proceeded as in the semi‐natural experiment. At 7 dpi, mildew‐infected leaves were inoculated with either *Ampelomyces* as a 70 ± 2 μl spray of hyperparasite spores in filter‐sterilised H_2_O at 1 × 10^6^ spore per ml, or the same volume of filter‐sterilised H_2_O. Mildew growth and sporulation was scored at 8 and 14 dpi on a categorical scale based on (Bevan, Crute & Clarke, 1993): 0: no growth, 1: few hyphae visible under microscope, 1.5: mycelial mass visible under microscope, 2: mycelia visible to naked eye and little or no sporulation visible under microscope, 3: abundant sporulation and lesion size <0.5 cm^2^, 4: abundant sporulation and lesion size >0.5 cm^2^. Each treatment in this *G* ^pathogen^ × *G* ^host^ × hyperparasite^+/−^ design was replicated 32 times, failed pathogen germinations (scored as a ≤1 on the above scale) by 14 dpi were trimmed from the data, as there is no host for the hyperparasite to infect. Final sample sizes ranged from 10 to 23 (mean 19) per treatment.

In a follow‐up experiment, we explored the effect of the hyperparasite on a later life‐history stage of the mildew. We followed the procedure as above with the same five pathogen strains but on a single host background (*Plant_4*). We inoculated these with either the hyperparasite or mock control at 8 dpi, and then screened for the presence/absence of mature chasmothecia at 14 dpi (present if ≥10 tan brown chasmothecia were observed). Each treatment combination (10) was replicated 26–60 times.

In both experiments, we omitted leaves which were inoculated with *Ampelomyces* but did not exhibit visible signs of infection by 14 dpi, because we cannot distinguish between a successful resistance response by the mildew or a germination failure by the hyperparasite.

### 2.4 Data analyses

Analyses were conducted in r as linear or generalised linear models. Minimum adequate models were derived through stepwise model simplification based on likelihood ratio tests and AIC values (Crawley, 2013). Overdispersion was tested and accounted for by fitting an observation‐level random effect in mixed models.

The effect of hyperparasite infection on within‐host–pathogen development in the field was modelled as the number of infected leaves on each source plant as a negative‐binomial response variable with *Ampelomyces* treatment as a fixed independent variable.

We analysed the proportion of recipient plants that became infected in the field with a binomial GLMM, where hyperparasite treatment of the source plant, genotype of the recipient plant and distance of the recipient from the source were included as fixed factors and pathogen infection severity of the source plant as a covariate. The identity of the source plant was included as a random effect to account for the block effect of eight recipient plants surrounding each source.

Infection severity on each recipient plant was analysed as the number of infected leaves on the plant at the end of the experiment with a negative‐binomial GLMM with the same starting model structure as described above. The number of infected leaves rather than the proportion of infected leaves was used to avoid biased infection load estimates due to the two plant genotypes inherently producing different numbers of leaves (*Plant_1*: 36.8 ± 0.9, *Plant_3*: 25.7 ± 1.3).

In the first laboratory experiment, the change in Bevan score between 8 and 14 dpi was modelled as the response variable, with pathogen genotype, plant strain, hyperparasite treatment/control, and all possible interactions as fixed effects. We initially included the petri dish that a given leaf was kept in as a random effect to account for four leaves from the same treatment in the same dish. However, AIC comparisons and assessment of residuals indicated that removing this gave a better model fit. For the second experiment, the proportion of mildew‐infected leaves that produced chasmothecia was modelled as a binomial variable with a logit link. The model structure was the same as above but without plant genotype, as only a single clonal line was used.

## 3 RESULTS

### 3.1 The importance of host resistance versus hyperparasite infection in the field

#### 3.1.1 Within‐host pathogen growth in field conditions

Hyperparasite infection had no significant effect on the number of leaves that became infected on source plants kept in semi‐natural field conditions (*χ* ^2^ = 1.32, *p *= 0.25).

#### 3.1.2 Pathogen dispersal and establishment

**Figure 2:**
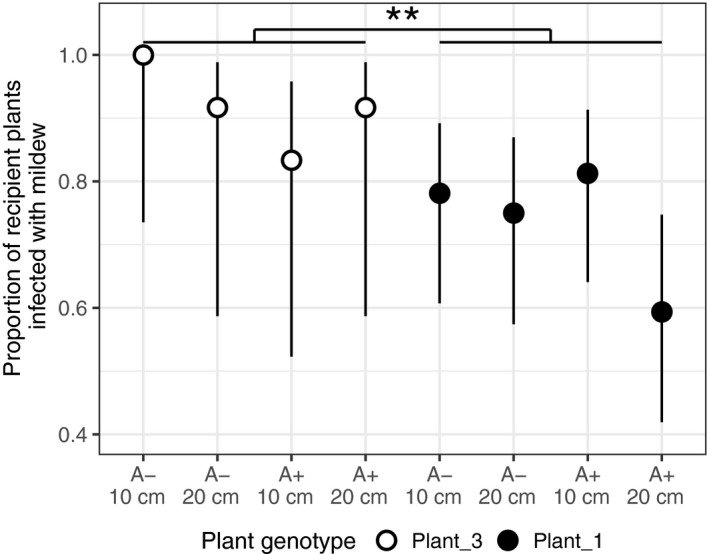
Host genotype significantly alters pathogen establishment (open vs. filled points), but hyperparasite infection of the source plant (A+ or A−) and distance of the recipient plant from the source plant (10 or 20 cm) have no significant effect. Bars = 95% CI with logit link

Recipient plant genotypes differed significantly in their probability of becoming infected with *Po. plantaginis* (*χ* ^2^ = 6.097, *df* = 1, *p *= 0.014, Figure 2). However, there was no significant effect of the recipient's distance from the source (*χ* ^2^ = 2.249, *df* = 1, *p *= 0.134), nor whether the source plant was hyperparasitised by *Ampelomyces* on the probability of infection (*χ* ^2^ = 1.279, *df* = 1, *p *= 0.258).

Infection severity on infected recipient plants was significantly, negatively correlated with the recipient's distance from the source plant (*χ* ^2^ = 4.7886, *df* = 1, *p *= 0.029), but recipient plant genotype had no significant effect (*χ* ^2^ = 2.012, *df* = 1, *p *= 0.156). *Ampelomyces* infection at the source had no significant impact on infection severity on recipients (*χ* ^2^ = 0.099, *df* = 1, *p *= 0.755).

Hyperparasite infection was found on only two leaves from two recipient plants. All source plants that were inoculated with the hyperparasite became infected with *Ampelomyces*, while none of the leaves from control source plants screened positively for the hyperparasite. Thus, cross‐contamination among source plants is negligible.

### 3.2 Hyperparasite and host genotype impact on pathogen growth and sporulation

**Figure 3:**
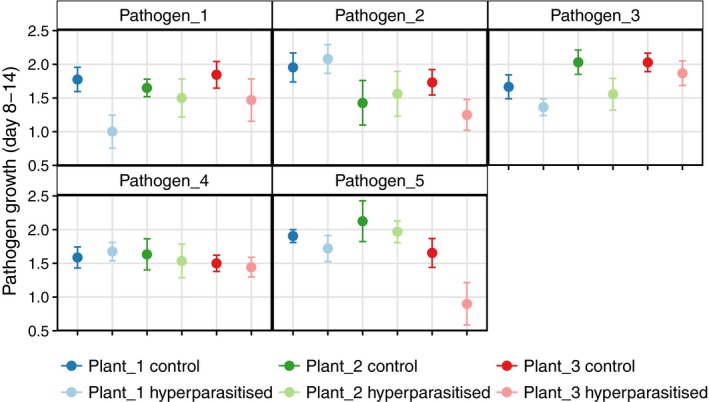
The interaction between host‐plant and pathogen genotype (bold points) significantly predicted pathogen growth between 8 and 14 days post inoculation under laboratory conditions (*p *= 0.003). Infection with *Ampelomyces* (pale points) had a significant, negative effect on pathogen growth (*p* = 0.039). Errors = SEM. There was no statistically significant interaction between hyperparasite infection and either plant or pathogen genotype

We found no significant three‐way interaction between pathogen strain, plant strain and *Ampelomyces* treatment on pathogen growth rate between 8 and 14 dpi (*F* = 0.863, *p = *0.545), nor any significant two‐way interactions between hyperparasite infection and pathogen strain (*F* = 1.367, *p = *0.244) or hyperparasite infection and plant genotype (*F* = 0.409, *p = *0.665). As a main term, *Ampelomyces* treatment significantly decreased the growth rate of powdery mildews compared with uninfected controls (*F* = 4.3, *df* = 1, *p = *0.039, Figure 3: bold vs. pale points), albeit this effect is small (10.39 ± 5.01% decrease in growth compared with the controls). In accordance with the known genotype × genotype nature of this system, the interaction between mildew and host‐plant genotype significantly affected mildew growth (*F* = 2.976, *df* = 8, *p *= 0.003). This is also true when we analyse only hyperparasite‐free controls; sporulation level of all five mildew genotypes at 14 dpi was significantly influenced by the interaction between host‐plant and pathogen genotypes (*G* ^Pathogen^ × *G* ^host^: *χ* ^2^ = 26.53, *df* = 10, *p *= 0.003).

**Figure 4:**
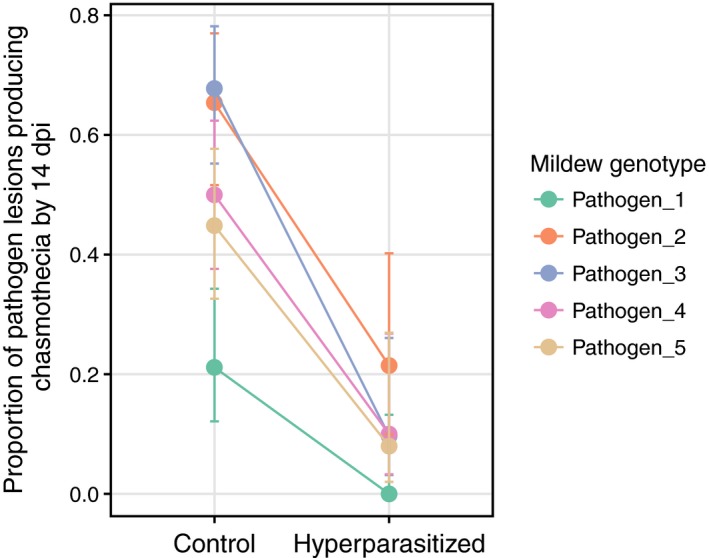
The probability of a pathogen producing overwinter resting structures (chasmothecia) by 14 dpi was significantly negatively affected by infection with *Ampelomyces* hyperparasites. Pathogen genotypes also significantly varied in their probability of producing resting structures, but there was no significant interaction between these main effects. Errors = 95% CI calculated with logit link

The second laboratory study found a significant reduction in the log‐odds (−1.14) of mildew lesions producing chasmothecia by 14 dpi when infected with *Ampelomyces* (*χ* ^2^ = 35.508, *df* = 1, *p *< 0.001, Figure 4). Pathogen genotypes significantly varied in their probability of producing chasmothecia (*χ* ^2^ = 55.403, *df* = 4, *p *< 0.001). There was no significant interaction between these factors (*χ* ^2^ = 6.96, *df* = 4, *p *= 0.138).

## 4 DISCUSSION

To our knowledge, this is the first study of the establishment and transmission of a pathogen simultaneously under bottom‐up control by host resistance, and top‐down control by hyperparasite attack. Yet, most pathogens are likely to face these two impacts in nature, and this scenario forms the basis of many biocontrol strategies for agricultural pests. We found that host genotype and the distance from disease foci were the overriding factors governing pathogen transmission and establishment during the onset of powdery mildew epidemics under semi‐natural conditions. We did not find any effect of hyperparasitism at the focal point of infection on either within‐host growth or between host transmission of *Po. plantaginis* during these early, critical stages of the mildew's life history. Our results confirm previous findings that host genotype and spatial structure can strongly influence pathogen growth and transmission (Laine, 2004; Tack et al., 2014). We then tested if hyperparasitism affected initial lesion development across different combinations of host–pathogen genotypes under laboratory conditions. On average, hyperparasite infection significantly reduced pathogen growth but, although the three‐way interaction was not significant, our data suggest that this varied among host–pathogen genotype combinations. Indeed, infection with the hyperparasite even had a positive effect in some cases. Finally, we explored the effect of the hyperparasite on later pathogen life‐history stages, which typically manifest after host resistance failed. Here, hyperparasitism significantly reduced the production of overwintering structures. This may explain why previous studies have found a link between hyperparasitism and powdery mildew extinction risk in nature (Tollenaere et al., 2014). Combined, our results suggest that top‐down control of pathogens by natural enemies may be sensitive to both the combination of pathogen and host genotype, and the specific pathogen life‐history stage that is targeted. In powdery mildew systems, bottom‐up control is likely to be the overriding determinant of early‐epidemic disease dynamics, while hyperparasitism affects later stages.

Our finding that any top‐down control by the *Ampelomyces* hyperparasite is highly context dependant is not entirely surprising. There is generally a paucity of evidence to demonstrate that infectious agents at any trophic level can exert strong, consistent top‐down influence to regulate the population dynamics of their hosts in nature. Rather, infectious disease epidemics are either too small to affect population dynamics or too severe but infrequent to consistently regulate host populations (Hall, Becker, Duffy & Cáceres, 2011). This may explain the necessity for hosts to evolve strong resistance strategies, rather than to rely on hyperparasites to ameliorate disease risk. Where parasite‐mediated population regulation has been observed, the effects are often either indirect (Holdo et al., 2009) or influenced by additional environmental factors. For example, while evidence suggests that nematode (*Trichostrongylus tenuis*) infections influence population cycling in Red Grouse (Hudson, Dobson & Newborn, 1998), this has been shown to function only in conjunction with other environmental factors that influence grouse population growth (Redpath et al., 2006). Similarly, cycling in Reindeer populations are influenced, in part, by the prevalence of gut nematode infections, but also strongly affected by winter rainfall (Albon et al., 2002). On the other hand, strong pathogen effects and high growth rates detected in stable laboratory studies may be attenuated by fluctuating variation in natural environments. Indeed, pathogen development rates can be directly influenced by abiotic conditions, and pathogen genotypes may respond differently to environmental variation (Mitchell et al., 2005; Parratt, Numminen & Laine, 2016; Price et al., 2004; Wolinska & King, 2009).

Our laboratory inoculation study suggests that for some pathogen genotypes, the hyperparasite can still reduce growth rate and can significantly reduce the production of the mildew's overwinter resting spores. Thus, we do not fully discount top‐down hyperparasite control as a fitness cost for this pathogen. Hyperparasitism in this system may be a secondary regulatory factor, whose effects are either too marginal to detect with our methods, or would be more severely felt under environmental conditions not tested here. Further, we find little spread of the hyperparasite within plants in our semi‐natural experiment, even though our design introduces *Ampelomyces* at the original focal infection and allows the hyperparasite enough time (20 days) to sporulate and disperse by rain splash as it does so in nature (Kiss et al., 2004). This suggests that hyperparasite transmission may lag behind the mildew's growth and not fully influence pathogen fitness until after the mildew has reached an epidemic peak. Thus, top‐down regulation of mildew populations may only occur during later‐epidemic stages and overwintering, rather than during the early onset that was predominantly tested here. Furthermore, in this study, we only present allopatric combinations of pathogen and hyperparasites collected from the same limited geographical area of 50 × 70 km, to control for any effect of local adaptation. In a previous study, we detailed that the outcome of hyperparasite infection can be significantly controlled by a specific genotype × genotype interactions (Parratt et al., 2017), so further exploration of any top‐down control with sympatric as well as allopatric combinations may reveal a stronger effect.

We find that host genotype and spatial structure are important determinants of within‐host–pathogen growth and transmission among host individuals during onset of *Po. plantaginis* epidemics. Our findings support previous work on the *Plantago‐Podosphaera* system, which shows that bottom‐up resistance strongly influences infection outcome (Laine, 2004) and that spatial structuring of host plants, even at very local scales (<2 m), impacts pathogen spread and establishment (Tack et al., 2014). Here, we show that the influence of these factors remain robust in the face of top‐down influence by the pathogen's natural enemy. While host resistance is liable to break down in the face of pathogen adaptation, in natural systems the standing diversity in resistance and the evolution of novel resistance types are likely to be effective strategies in preventing disease epidemics (Jousimo et al., 2014; Laine et al., 2011). Indeed, bottom‐up disease control has proven effective against many pathogens that threaten human health and food production, and is at the core of crop resistance breeding and vaccination programmes. Further research is needed to determine whether resistance breakdown following pathogen adaptation observed in crop systems could be prevented by simultaneous employment of top‐down and bottom‐up control. Specifically, whether targeting specific life‐history phases of pathogens with top‐down biocontrol might negate the need for resistance to be effective year‐in‐year‐out.

In this study, we attempted to capture some of the biological reality faced by pathogens in nature, by exposing them to two challenges: host resistance and natural enemy attack. Furthermore, we explore these influences in both the laboratory and semi‐natural settings, thus accounting for some of the variation under which these processes will operate in nature. Our data give insight into unconsidered ecological interactions among infective agents, and their impacts upon natural disease spread. We find that bottom‐up control by host resistance takes the front‐seat in determining pathogen growth and transmission during the early establishment of infections and during the onset of epidemics in this system. The physical distance of susceptible hosts also plays an important role in governing the severity of disease outbreaks during this critical phase. In contrast, the effect of hyperparasitism is likely to be felt most acutely during later stages of the powdery mildew life cycle. The importance of top‐down versus bottom‐up control is a longstanding debate in ecology, and our results offer novel insight into how their relative importance varies across pathogen epidemics. This finding has far‐reaching implications for successful disease control and management efforts, suggesting that the use of hyperparasites as biocontrol measures may be highly sensitive to the timing of their application, and to the life‐history stages of both pathogen and hyperparasite.

## AUTHORS' CONTRIBUTIONS

S.R.P. and A.‐L.L. conceived and designed the study. S.R.P. collected and analysed the data. S.R.P. and A.‐L.L. wrote the manuscript and approved the final version.

## DATA ACCESSIBILITY

Data available via the Dryad Digital Repository https://doi.org/10.5061/dryad.79vg007 (Parratt & Laine, 2018).
