# Supplementary material for: Pathogen dynamics under both bottom‐up host resistance and top‐down hyperparasite attack
Source: J Appl Ecol. 2018 Jun 19;55(6):2976–85. doi: 10.1111/1365-2664.13185 (PMC6220889; doi:10.1111/1365-2664.13185)
Supplement: Supplementary file 1 [file JPE-55-2976-s001.docx]

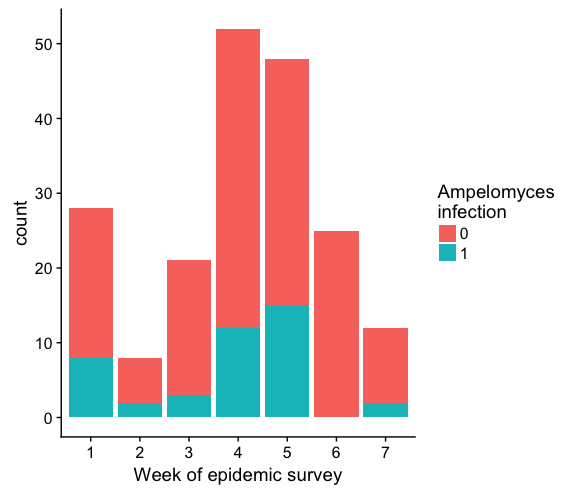


**Figure S1:** Field surveys of natural epidemics in 15 populations of *Podosphaera plantaginis* in the Åland Islands (SW Finland) found evidence of *Ampelomyces* infection during the establishment and onset phases. Bars are counts of genetic samples taken from infected plants on the week they were first found to be infected with mildew. qPCR screening was used to screen these samples for presence (1) or absence (0) of the hyperparasite. Sampling methods described in detail in Parratt *et al* 2017. Established *Ampelomyces* infections are detectable on infected plants during the first week of sampling, most likely due to the hyperparasite overwintering within the vicinity of the powdery mildew, and re-establishing hyperparasitism upon pathogen germination in the spring/summer.
