# Supplementary material for: Pathogen dynamics under both bottom‐up host resistance and top‐down hyperparasite attack
Source: J Appl Ecol. 2018 Jun 19;55(6):2976–85. doi: 10.1111/1365-2664.13185 (PMC6220889; doi:10.1111/1365-2664.13185)
Supplement: Supplementary file 2 [file JPE-55-2976-s002.docx]

**Table S1:** Designations and origins of organisms used in the laboratory and field studies.

| Organism | Strain ID | Numerical strain ID | Population of origin | Year isolated |
| --- | --- | --- | --- | --- |
| *Plantago lanceolata*  (host plant) | *Plant_1* | 511_11 | 511 | 2010 |
|  | *Plant_2* | 2220_M1 | 2220 | 2010 |
|  | *Plant_3* | 4_13 | 4 | 2010 |
|  |  |  |  |  |
| *Podosphaera plantaginis*  (Powdery mildew pathogen) | *Path_1* | 01A | 705 | 2013 |
|  | *Path_2* | 12A | 1556 | 2013 |
|  | *Path_3* | 29A | 3117 | 2013 |
|  | *Path_4* | 33B | 1097 | 2013 |
|  | *Path_5* | 40A | 5509 | 2013 |
|  |  |  |  |  |
| *Ampelomyces spp* | 294_11 | 294_11 | 294 | 2014 |
