# Supplementary material for: Pathogen dynamics under both bottom‐up host resistance and top‐down hyperparasite attack
Source: J Appl Ecol. 2018 Jun 19;55(6):2976–85. doi: 10.1111/1365-2664.13185 (PMC6220889; doi:10.1111/1365-2664.13185)
Supplement: Supplementary file 3 [file JPE-55-2976-s003.docx]

**Table S2:** Length of inoculated leaves and total number of leaves on focal plants. Initially, 75 focal plants were grown, from these we selected 22 that were in good condition and hosted visible powdery mildew symptoms on both inoculated leaves at 25 dpi at the onset of the experiment.

| Focal plant ID | Nb leaves before inoculation | 1^st^ Inoculated leaf (mm) | 2^nd^ inoculated leaf (mm) | Nb leaves at time of sampling | *Ampelomyces* treatment (Yes/No) |
| --- | --- | --- | --- | --- | --- |
| F44 | 18 | 131 | 144 | 43 | N |
| F75 | 13 | 137 | 167 | 29 | Y |
| F14 | 19 | 137 | 136 | 29 | Y |
| F35 | 14 | 118 | 127 | 58 | Y |
| F27 | 14 | 109 | 151 | 32 | Y |
| F23 | 10 | 113 | 116 | 33 | Y |
| F63 | 26 | 125 | 133 | 49 | Y |
| F40 | 12 | 122 | 135 | 39 | Y |
| F58 | 13 | 110 | 116 | 38 | Y |
| F36 | 10 | 118 | 120 | 27 | Y |
| F60 | 18 | 106 | 115 | 48 | Y |
| F57 | 11 | 140 | 138 | 37 | Y |
| F11 | 14 | 146 | 147 | 33 | N |
| F46 | 19 | 114 | 121 | 24 | N |
| F70 | 11 | 137 | 141 | 33 | N |
| F32 | 12 | 118 | 147 | 29 | N |
| F8 | 9 | 131 | 126 | 22 | N |
| F45 | 18 | 138 | 143 | 40 | N |
| F55 | 16 | 121 | 125 | 47 | N |
| F41 | 15 | 127 | 158 | 35 | N |
| F48 | 14 | 114 | 125 | 31 | N |
| F31 | 21 | 120 | 131 | 34 | N |
