# Supplementary material for: Pathogen dynamics under both bottom‐up host resistance and top‐down hyperparasite attack
Source: J Appl Ecol. 2018 Jun 19;55(6):2976–85. doi: 10.1111/1365-2664.13185 (PMC6220889; doi:10.1111/1365-2664.13185)
Supplement: Supplementary file 4 [file JPE-55-2976-s004.docx]

**Appendix S1: Supplementary methods & references**

Plant, pathogen and hyperparasite isolation and culture

All organisms used in this stud were isolated from the metapopulation of *Po. plantaginis* in the Åland archipelago (SW Finland). To avoid confounding effects of local adaptation, all plant, pathogen, and hyperparasite isolates are allopatric (isolated from different subpopulations - see supplementary Table S1). Clonal lines of *Pl. lanceolata* (*Plant_1, Plant_2* and *Plant_3*,) were isolated as seeds and grown as root clones in 1:1 mix of potting soil and sand under greenhouse conditions (20±2^o^C, 18h:6h light:dark or ambient Helsinki summertime day length). All *Po. plantaginis* isolates (*Path_1, Path_2, Path_3, Path_4* and *Path_5*) were cultured on detached leaves from broadly susceptible plants on wet filter paper discs in Ø 9cm petri dishes. Mildew were transferred to fresh leaves every 14 days using sterile paintbrushes as described in (Laine 2004; Susi & Laine 2013). The *Ampelomyces* isolate was maintained on cell free media (3g NaNO_3_, 1g K_2_HPO_4_, 0.5g KCL, 0.5g MgSO_4_, 30g C_12_H_22_O_11_, 115g Agar & Barley malt extract litre^-1^) and turned over ever 4-6 weeks. Mildew strains and the hyperparasite were isolated as infected leaves during September in their respective years. Mildews are taken as single conidial chains and passaged this way on detached *Pl. lanceolata* leaves under laboratory conditions for at least three sporulation cycles to ensure that they are pure single strains. *Ampelomyces* was isolated as mature pycnidia from the surface of field collected leaves onto culture media containing 3ml/litre of Chloramphenicol (0,1g/ml) and identified as the correct fungus based on morphology and characteristic slow growth (Szentivanyi *et al.* 2005).

## Screening for hyperparasite

To ensure that the hyperparasite established on the inoculated source we used a qPCR detection technique to quantify the number of leaves supporting *Ampelomyces* on each focal and satellite plant. DNA was extracted using the E.Z.N.A™ plant kit (Promega) according to the manufacturer’s instructions. The qPCR technique amplifies an *Ampelomyces* specific region of the ITS ribosomal RNA using the primers AmpITS_F (GCTGCCAATTGCTTTGAGAT) and AmpITS_R (GATGAAGAACGCAGCGAAAT) as reported in (Tollenaere *et al.* 2014). Briefly: 1μl of sample template was added to 5μl SYBRGreen qPCR Master Mix, 0.5μl of each primer at 5μM and 4μl of dH_2_O to make a 10μl total reaction volume. Samples were initially denatured for 10 minutes at 95^o^C, and then run for 30 cycles of 30s at 95^o^C, 30s at 60 ^o^C and 30s at 72 ^o^C in a Bio-Rad CFX384 thermocycler. This was followed by a melting curve analysis from 45 ^o^C to 95 ^o^C by 0.5 ^o^C increment every 5s. Samples with a mean CQ value (the cycle at which target sequence amplification is detectable) ≤24 (over three replicate reactions with SD < 0.2) were determined to possess an established *Ampelomyces* infection. The reliability of this threshold as a detection method has previously been experimentally demonstrated (Tollenaere *et al.* 2014).

**References**

Laine, A.-L. (2004) Resistance Variation within and among Host Populations in a Plant: Pathogen Metapopulation: Implications for Regional Pathogen Dynamics. *Journal of Ecology*, **92**, 990–1000.

Parratt, S.R., Barres, B., Penczykowski, R.M. & Laine, A.-L. (2017) Local adaptation at higher trophic levels: Contrasting hyperparasite-pathogen infection dynamics in the field and laboratory. *Molecular Ecology*, **26**, 1964–1979.

Susi, H. & Laine, A.-L. (2013) Pathogen life-history trade-offs revealed in allopatry. *Evolution*, **67**, 3362–3370.

Szentivanyi, O., Kiss, L., Russell, J.C., Kovács, G.M., Varga, K., Jankovics, T., Lesemann, S., Xu, X.-M. & Jeffries, P. (2005) Ampelomyces mycoparasites from apple powdery mildew identified as a distinct group based on single-stranded conformation polymorphism analysis of the rDNA ITS region. *Mycological Research*, **109**, 429–438.

Tollenaere, C., Pernechele, B., Mäkinen, H.S., Parratt, S.R., Németh, M.Z., Kovács, G.M., Kiss, L., Tack, A.J.M. & Laine, A.-L. (2014) A hyperparasite affects the population dynamics of a wild plant pathogen. *Molecular Ecology*, **23**, 5877–5887.
